# TMPRSS11D and TMPRSS13 Activate the SARS-CoV-2 Spike Protein

**DOI:** 10.3390/v13030384

**Published:** 2021-02-28

**Authors:** Mai Kishimoto, Kentaro Uemura, Takao Sanaki, Akihiko Sato, William W. Hall, Hiroaki Kariwa, Yasuko Orba, Hirofumi Sawa, Michihito Sasaki

**Affiliations:** 1 Division of Molecular Pathobiology, Research Center for Zoonosis Control, Hokkaido University, N20 W10, Kita-ku, Sapporo 001-0020, Japan; kishimoto@czc.hokudai.ac.jp (M.K.); orbay@czc.hokudai.ac.jp (Y.O.); h-sawa@czc.hokudai.ac.jp (H.S.); 2 Drug Discovery and Disease Research Laboratory, Shionogi & Co., Ltd., 3-1-1 Futaba-cho, Toyonaka, Osaka 561-0825, Japan; kentaro.uemura@shionogi.co.jp (K.U.); takao.sanaki@shionogi.co.jp (T.S.); akihiko.sato@shionogi.co.jp (A.S.); 3 Division of Anti-Virus Drug Research, Research Center for Zoonosis Control, Hokkaido University, N20 W10, Kita-ku, Sapporo 001-0020, Japan; 4 Laboratory of Biomolecular Science, Faculty of Pharmaceutical Sciences, Hokkaido University, N12 W6, Kita-ku, Sapporo 060-0812, Japan; 5 National Virus Reference Laboratory, School of Medicine, University College Dublin, DO4V1W8 Dublin, Ireland; william.hall@ucd.ie; 6 Centre for Research in Infectious Diseases, School of Medicine, University College Dublin, DO4V1W8 Dublin, Ireland; 7 International Collaboration Unit, Research Center for Zoonosis Control, Hokkaido University, N20 W10, Kita-ku, Sapporo 001-0020, Japan; 8 Global Virus Network, 725 West Lombard St, Room S413, Baltimore, MD 21201, USA; 9 Laboratory of Public Health, Faculty of Veterinary Medicine, Hokkaido University, N18 W9, Kita-ku, Sapporo 060-0818, Japan; kariwa@vetmed.hokudai.ac.jp

**Keywords:** severe acute respiratory syndrome-like coronavirus-2 (SARS-CoV-2), type II transmembrane serine protease (TTSP), spike protein

## Abstract

Severe acute respiratory syndrome coronavirus-2 (SARS-CoV-2) utilizes host proteases, including a plasma membrane-associated transmembrane protease, serine 2 (TMPRSS2) to cleave and activate the virus spike protein to facilitate cellular entry. Although TMPRSS2 is a well-characterized type II transmembrane serine protease (TTSP), the role of other TTSPs on the replication of SARS-CoV-2 remains to be elucidated. Here, we have screened 12 TTSPs using human angiotensin-converting enzyme 2-expressing HEK293T (293T-ACE2) cells and Vero E6 cells and demonstrated that exogenous expression of TMPRSS11D and TMPRSS13 enhanced cellular uptake and subsequent replication of SARS-CoV-2. In addition, SARS-CoV-1 and SARS-CoV-2 share the same TTSPs in the viral entry process. Our study demonstrates the impact of host TTSPs on infection of SARS-CoV-2, which may have implications for cell and tissue tropism, for pathogenicity, and potentially for vaccine development.

## 1. Introduction

Coronavirus disease 2019 (COVID-19) has emerged as a pandemic and poses a significant public health threat despite its often low morbidity and mortality rate in certain geographic locations. To date, >100,000,000 people have been infected, resulting in >2,100,000 deaths globally (https://coronavirus.jhu.edu/map.html, accessed on 4 January 2021). Infection is caused by a novel severe acute respiratory syndrome coronavirus-2 (SARS-CoV-2), which is closely related to SARS-CoV-1 [1,2]. As with SARS-CoV-1, SARS-CoV-2 mainly infects the respiratory tract and is primarily transmitted via the respiratory route, causing respiratory illness that can partially progress to severe pneumonia [3]. In addition, gastrointestinal illness—possibly via a faecal-oral transmission route—was also observed in SARS-CoV-2 patients [4,5,6]. Moreover, it has been pointed out that the central nervous system (CNS) can be involved in SARS-CoV-2 infection, and this may also contribute to respiratory failure [7,8]. However, the specific cellular and tissue tropisms and pathology of SARS-CoV-2 remains to be further clarified.

Host cell factors involved in the viral entry steps are major determinants of coronavirus tropism and efficiency of cellular entry. SARS-CoV-2 enters into cells in the following steps: i) Virion of SARS-CoV-2 attaches to the target cell by interaction between the S1 subunit of the spike (S) protein and its cognate receptor, angiotensin-converting enzyme 2 (ACE2), ii) the binding of the S protein to ACE2 provokes conformational change of the S protein to a pre-fusion state, iii) the S2 subunit of the S protein is cleaved by host proteases at the S2′ site to trigger irreversible refolding of the S2 subunit into a post-fusion conformation, and iv) fusion of the cell/viral membranes occurs to introduce the viral genome into the cytosol of the host cell [9,10,11,12]. While SARS-CoV-1 requires cleavage of the S protein at the S1/S2 site by host proteases in the entry steps, the S1/S2 site of the S protein of SARS-CoV-2 is cleaved by intracellular protease furin in the viral assembly step, which may affect the cell tropism and the entry efficiency of SARS-CoV-2 [13]. In addition, it is suggested that the stability and glycosylation state of the S protein regulates its conformations to maintain the contact with ACE2 receptor, which is also related to vital entry efficiency [14,15,16].

As with SARS-CoV-1, SARS-CoV-2 utilizes two different entry pathways which involve different host proteases for S2 subunit cleavage: a type II transmembrane serine protease (TTSP)-dependent pathway, and a cathepsin B/L-dependent pathway [17,18]. In the former route, the S2 subunit is cleaved by TTSPs, including plasma membrane-associated transmembrane protease, serine 2 (TMPRSS2) on the cell surface, and mediates direct fusion of the viral envelope with the cellular membrane [9,17,18]. In the latter route, the virions of SARS-CoV-2 are taken into an endosome, and then the S2 subunit is cleaved by lysosomal protease cathepsin B/L [17,18]. While SARS-CoV-2 exclusively depends on cathepsin B/L for its S2 subunit activation in some TTSP-deficient cell lines, TTSP-dependent activation enhances the spread of SARS-CoV-2 in TTSP-expressing cells [19].

Human TTSPs consist of four subtypes and 18 members: the hepsin/TMPRSS subfamily (including TMPRSS1-5, 12, 13 and 15), matriptase subfamily (including TMPRSS6, 7, 9 and 14), corin subfamily (including TMPRSS10), and HAT/DESC subfamily (including TMPRSS11A, 11B, 11D, 11E, and 11F) [20]. TMPRSS2 is a well-characterized TTSP which serves as a host factor involved in the replication of certain viruses. In addition to TMPRSS2, the relationship between other TTSP families and viral life cycles, particularly influenza viruses and coronaviruses, have been extensively investigated [21,22,23,24,25,26,27]. For SARS-CoV-1, TMPRSS2, 11A, 11D, 11E, and 13 were shown to activate the S protein [25,26,27]. For SARS-CoV-2, TMPRSS2, 4, 11A, 11D, and 11E all activate the S protein and enhance S-mediated cell fusion in HEK293 cells expressing human ACE2 (hACE2) [28,29]. It has also been demonstrated that TMPRSS4 promotes SARS-CoV-2 infection in cooperation with TMPRSS2 in human small intestinal enterocytes [30]. However, previous reports have essentially been limited to the detection of SARS-CoV-2 S protein activation, and only a small number has involved functional analysis using infectious SARS-CoV-2. In the present study, we have screened the activity of 12 TTSPs using infectious SARS-CoV-2 and SARS-CoV-1 and characterized the role of TMPRSS11D and 13 on viral entry. Our findings demonstrate the potential ability of these TTSPs to enhance the SARS-CoV-2 life cycle, and this may have important implications for the cell and tissue tropisms and pathogenicity of the virus.

## 2. Materials and Methods

### 2.1. Cells and Viruses

HEK293T (293T) cells were grown in Dulbecco’s Modified Eagles Medium (DMEM) with high glucose (Sigma-Aldrich, St. Louis, MO, USA) with 10% fetal bovine serum (FBS). Vero E6 cells were grown in DMEM with 10% FBS and 100 U/mL penicillin and 100 μg/mL streptomycin. Vero E6 cells stably expressing human TMPRSS2 (Vero-T2 cells) and 293T cells stably expressing hACE2 (293T-ACE2 cells) were prepared as described previously [31]. Cells were cultured at 37 °C with 5% CO2. The SARS-CoV-2 isolated strain WK-521 and SARS-CoV-1 strain Hanoi were kindly provided by Drs. Saijyo, Shimojima (National Institute of Infectious Diseases, Tokyo, Japan) and Dr. Morita (Institute of Tropical Medicine Nagasaki University, Nagasaki, Japan), respectively, and propagated in Vero-T2 cells [32].

### 2.2. Plasmids and Transfection

The cDNAs of TMPRSS1 and 11A were synthesized as gBlocks Gene Fragments (Integrated DNA Technologies, Coralville, IA, USA). The cDNA clones of TMPRSS2, 11D, 11E, and 13 were obtained from DNAFORM (Yokohama, Japan). The cDNAs of TMPRSS3, 4, 5, 6, 10, and 14 were obtained by RT-PCR using total RNA from cell lines (Table S1). A total of 12 human TTSP genes were individually cloned into plasmid vector pCXSN—the pCMV derivative having the CMV promoter and a HA tag sequence at the C-terminus of the encoding sequences, prepared via XhoI/NotI or SalI/NotI restriction enzyme sites. The sequences of each cDNA clone were verified by sanger sequence (Table S1). For analysis of transient protease expression, 293T-ACE2 cells were seeded on 24-well plates at a density of 2.0×10^5^ cells/well, which were transiently transfected with plasmids encoding 12 TTSPs with the C-terminal HA tag or an empty plasmid as a control using Polyethylenimine Max (Polysciences, Inc., Warrington, PA, USA). Media were changed with fresh medium after 6 h post-transfection. At 48 h post-transfection, cells were used for immunoblotting assay and virus entry assays.

### 2.3. Generation of Vero E6 Cells Stably Expressing TTSPs

Human TMPRSS11D, 11E, and 13 genes were individually cloned into the self-inactivating lentiviral vector plasmid CSII-CMV-MCS-IRES2-Bsd, which was kindly provided by Dr. Miyoshi (RIKEN, Ibaraki, Japan). Lentivirus particles were prepared by co-transfection with the lentiviral vector plasmid and Lentiviral High Titer Packaging Mix (Takara Bio, Kusatsu, Japan), and then inoculated to Vero E6 cells with 10 μg/mL of polybrene. Transduced cells were selected in the presence of 10 μg/mL of blasticidin-S (FUJIFILM Wako, Osaka, Japan).

### 2.4. Immunoblotting

Cells were lysed in lysis buffer [1% NP-40, 20 mM Tris-HCl (pH 7.5), 150 mM NaCl, 5 mM EDTA] supplemented with a cOmplete ULTRA protease inhibitor cocktail (Roche Diagnostics, Mannheim, Germany). Cell lysates were separated by SDS-PAGE and transferred onto Immobilon-P PVDF membranes (Merck, Burlington, MA, USA). For detection of HA-tagged TTSPs which were transiently expressed in 293T-ACE2 cells, blotted membranes were incubated in HRP-conjugated anti-HA tag antibody (H6533, Sigma-Aldrich) diluted with 5% skim milk in TBS-T buffer [25 mM Tris-HCl (pH 7.5), 137 mM NaCl, 2.7 mM KCl]. For detection of TTSP-expression in Vero E6 cells, blots were incubated with primary antibodies: anti-TMPRSS2 (ab92323, Abcam, Cambridge, UK) antibody diluted with 5% skim milk in TBS-T buffer and anti-TMPRSS11D (GTX117370, GeneTex, Irvine, CA, USA), anti-TMPRSS11E (PA5-48775, Invitrogen, Waltham, MA, USA) and anti-TMPRSS13 (GTX117425, GeneTex) antibodies in Signal Booster (Beacle, Kyoto, Japan). The blots were then incubated with HRP-conjugated secondary antibodies. The HRP-conjugated anti-β-actin antibody (PM053-7, MBL, Nagoya, Japan) was used as a loading control. Signals were developed using Immobilon Western Chemiluminescent HRP Substrate (Merck).

### 2.5. Virus Entry Assay

Cells were incubated with SARS-CoV-1 or SARS-CoV-2 at a multiplicity of infections (MOI) of 1 in the presence of the cathepsin inhibitor, 25 μM E-64d (Abcam, Cambridge, MA, USA) or DMSO as control. After 1 h absorption, cells were washed with PBS and cultured in maintenance medium. At 4 h post-infection (hpi), total RNAs were extracted from inoculated cells using ISOGEN (Nippon Gene, Tokyo, Japan) and a Direct-zol RNA MiniPrep Kit (Zymo Research, Orange, CA, USA). Extracted RNAs were subjected to qRT-PCR analysis with the THUNDERBIRD Probe One-step qRT-PCR Kit (TOYOBO, Osaka, Japan). The sequences of primers and probes targeting the N gene of SARS-CoV-1 and SARS-CoV-2 have been described previously [33,34]. Human ACTB (Beta Actin) Endogenous Control (Applied Biosystems, Foster City, CZ, USA) and nonhuman primate β-actin were employed as endogenous controls [35].

### 2.6. Multi-Cycle Replication Assay

Vero-T11D, Vero-T11E, and Vero-T13 cells were seeded on 12-well plates at a density of 2.0×10^5^ cells/well and infected with SARS-CoV-1 and SARS-CoV-2 at MOI of 0.01. After 1 h incubation, cells were washed with PBS and cultured in fresh medium. At 24, 48, and 72 hpi, culture supernatants were collected and subjected to virus titration by plaque assay. For virus titration, Vero-T2 cells were inoculated with 10-fold serially diluted culture supernatants and then layered with DMEM containing 2% FBS and 0.5% agar. After 48 h incubation, cells were fixed with 3.7% buffered formaldehyde and stained with 1% crystal violet in absolute ethanol, and plaques were then manually counted.

### 2.7. Indirect Immunofluorescence Assay (IFA)

Vero E6, Vero-T2, Vero-T11D, Vero-T11E, and Vero-T13 cells were infected with SARS-CoV-2 at an MOI of 8. At 24 hpi, cells were fixed with 3.7% buffered formaldehyde. After treatment with ice-cold methanol, cells were stained with the primary antibody, anti-SARS-CoV-2 S antibody (GTX632604, GeneTex), and diluted with PBS buffer containing 1% Block Ace (KAC, Kyoto, Japan). Cells were then incubated with Alexa 488-conjugated anti-mouse IgG antibody (Invitrogen) in PBS buffer containing 1% Block Ace and 1% Hoechst 33342 (Invitrogen).

### 2.8. Statistical Analysis

One-way analysis of variance (ANOVA) with Dunnett’s test was used to determine statistical significance.

## 3. Results

### 3.1. Evaluation of the Effects of TTSPs on the Entry of SARS-CoV-1 and SARS-CoV-2 Using 293T-ACE2 Cells

**Figure 1:**
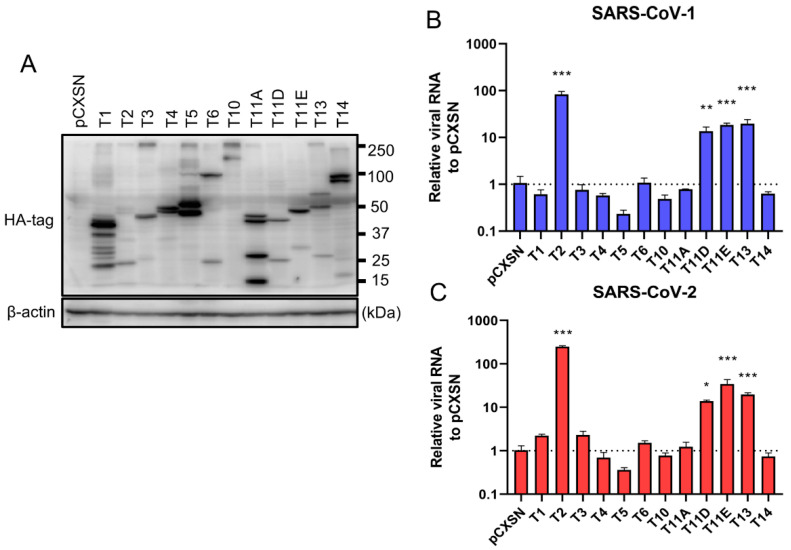
Entry of SARS-CoV-1 and SARS-CoV-2 in TTSP-expressing 293T-ACE2 cells. (**A**) Expression of TTSPs in 293T-ACE2 cells. Plasmids encoding TMPRSS1 (T1, 45 kDa), TMPRSS2 (T2, 53 kDa), TMPRSS3 (T3, 49 kDa), TMPRSS4 (T4, 48 kDa), TMPRSS5 (T5, 50 kDa), TMPRSS6 (T6, 89 kDa), TMPRSS10 (T10, 116 kDa), TMPRSS11A (T11A, 48 kDa), TMPRSS11D (T11D, 46 kDa), TMPRSS11E (T11E, 48 kDa), TMPRSS13 (T13, 61 kDa), TMPRSS14 (T14, 95 kDa), and empty plasmid pCXSN (as a control) with a C-terminal HA tag were transiently transfected into 293T-ACE2 cells. Protease expressions in cell lysates were detected by immunoblotting with an anti-HA antibody. Detection of β-actin was employed as a loading control. Similar results were obtained in three independent experiments. (**B**,**C**) Twelve types of TTSP-transfected 293T-ACE2 cells were infected with SARS-CoV-1 (**B**) and SARS-CoV-2 (**C**). Total RNAs were extracted from cells at 4 hpi and analyzed by qRT-PCR. Levels of N gene of SARS-CoV-1 and SARS-CoV-2 were normalized with that of β-actin mRNA. The values in the graphs are shown as means ± S.D. of triplicates. One-way ANOVA with Dunnett’ s test was used to determine the statistical significance compared to no-TTSPs controls; * *p <* 0.05, ** *p <* 0.01, *** *p* < 0.001. Data are representative of two independent experiments.

To investigate the role of TTSPs in addition to TMPRSS2 in the entry steps of both SARS-CoV-1 and SARS-CoV-2, 293T-ACE2 cells were transiently transfected with each of the 12 different TTSP-encoding plasmids and subjected to virus entry assays. The expressions of HA-tagged TTSPs were confirmed by immunoblotting (Figure 1A). Exogenous expressions of TTSPs were confirmed as bands of the expected molecular weight. In addition to bands of the predicted molecular weight, bands with smaller molecular weights were also observed, indicating autocatalytic activation of the TTSPs. Signals of full-length TMPRSS2 at 53 kDa and its cleaved form at 37 kDa were weaker than those of other TTSPs. SARS-CoV-2 employs two entry pathways—the direct fusion mode mediated by TTSP, and the endocytosis route mediated by cathepsin B/L [17,18]. To investigate the role of TTSPs on viral entry, the TTSP-expressing 293T-ACE2 cells were inoculated with either SARS-CoV-1 or SARS-CoV-2, and viral RNA levels at the early phase of infection were quantified using qRT-PCR (Figure 1B,C). The assay was conducted in the presence of E-64d, an inhibitor of cathepsin B/L, in order to block TTSP-independent viral entry. Expression of TMPRSS2, 11D, 11E, and 13 significantly increased viral RNAs isolated from viral inoculated cells at 4 hpi, indicating that these TTSPs enhanced entry of both SARS-CoV-1 and SARS-CoV-2 in the presence of E-64d. Among the tested TTSPs, TMPRSS2 exhibited the highest enhancement of activity for entry of SARS-CoV-1 and SARS-CoV-2. Based on these results, we further examined TMPRSS11D, 11E, and 13 in our subsequent experiments.

### 3.2. TMPRSS11D and 13 Facilitate SARS-CoV-2 Replication

**Figure 2:**
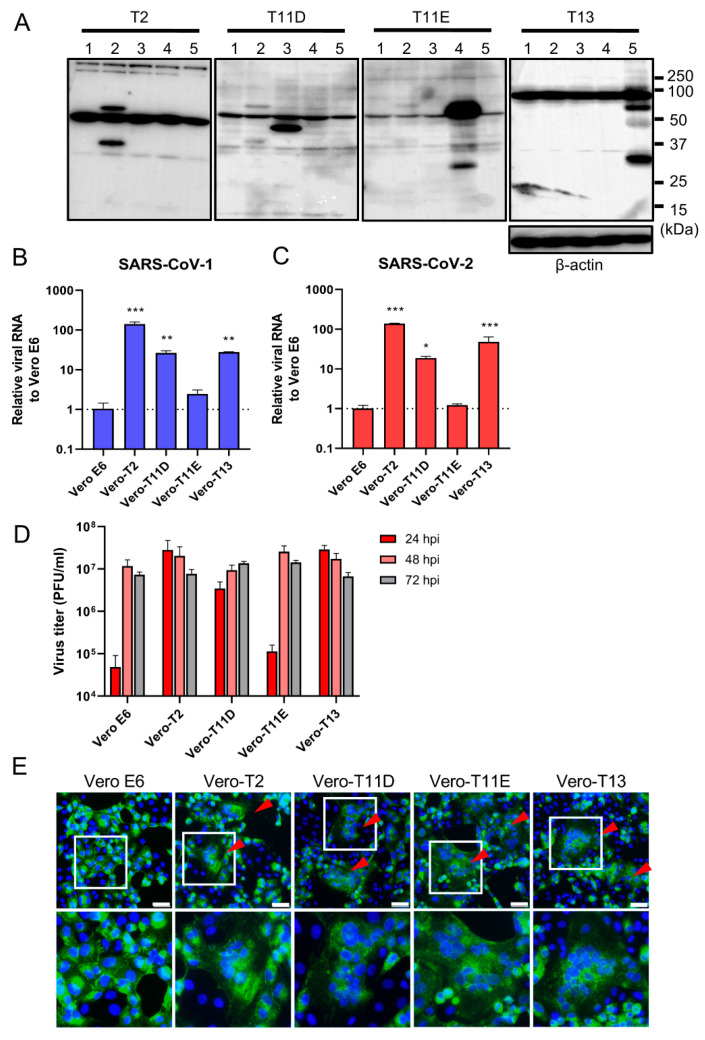
Effect of TTSPs on viral entry and replication in Vero-TTSP cells. (**A**) TTSPs’ expression in Vero E6 cells which were transduced with a lentiviral vector expressing TMPRSS2, 11D, 11E, and 13 was confirmed by immunoblotting; lane 1: Vero E6, lane 2: Vero-T2, lane 3: Vero-T11D, lane 4: Vero-T11E, lane 5: Vero-T13. (**B**,**C**) TTSP-transduced Vero E6 cells were infected with SARS-CoV-1 (**B**) and SARS-CoV-2 (**C**). Total RNAs were extracted from cells at 4 hpi and analyzed by qRT-PCR. Levels of N genes of SARS-CoV-1 and SARS-CoV-2 were normalized to that of β-actin mRNA. The values in the graphs are shown as means ± S.D. of triplicates. One-way ANOVA with Dunnett’ s test was used to determine the statistical significance compared to no-TTSPs controls; * *p* < 0.05, ** *p* < 0.01, *** *p* < 0.001. Data are representative of two independent experiments. (**D**) Vero E6, Vero-T2, T11D, T11E, and T13 cells were infected with SARS-CoV-2 (MOI=0.01). Culture supernatants were harvested at 24, 48, and 72 hpi and subjected to virus titration using plaque assays. The values in the graphs are shown as means ± S.D. of triplicates. (**E**) Fusion activity of SARS-CoV-2-infected cells. Vero E6, Vero-T2, T11D, T11E, and T13 cells were infected with SARS-CoV-2 (MOI=8). At 24 hpi, cells were fixed and stained with anti-SARS-CoV-2 spike antibody (green) and Hoechst 33342 (blue). Scale bars indicate 50 μm. Red arrowheads demonstrate cell syncytia. Data are representative of two independent experiments. Areas in white squares are magnified in lower panels.

We next established four different stably expressing TTSPs, including TMPRSS2 (Vero-T2 cells), 11D (Vero-T11D cells), 11E (Vero-T11E cells), and 13 (Vero-T13 cells) expressing Vero E6 cells which were highly susceptible to both SARS-CoV-1 and SARS-CoV-2 [9,13,19,36]. Immunoblotting analysis using each specific antibody to four TTSPs revealed the expression of each TTSP gene in the transduced cells, but not in parental Vero E6 cells (Figure 2A). Subsequently, Vero E6, Vero-T2, T11D, T11E, and T13 cells were subjected to virus entry assay using SARS-CoV-1 or SARS-CoV-2 in the presence of E-64d (Figure 2B,C). Cellular uptake of the viruses in Vero-T2, T11D, and T13 cells was significantly higher than in parental Vero E6 cells at 4 hpi, which was consistent with the results of transiently TTSPs-expressing 293T-ACE2 cells. However, no significant enhancement of viral entry was observed in Vero-T11E cells. These results suggest that not only TMPRSS2, but also TMPRSS11D and 13 enhance the entry of SARS-CoV-2, and that TMPRSS11D and 13 play roles in the entry steps of both SARS-CoV-1 and SARS-CoV-2.

To assess the impact of TTSPs on virus replication, Vero E6, Vero-T2, T11D, T11E, and T13 cells were infected with SARS-CoV-2, and the progeny virus in culture supernatants at 24, 48, and 72 hpi were titrated by plaque assay (Figure 2D). Infectious virus titers of supernatants in Vero-T2, T11D, and T13 were approximately 100-fold higher than that of Vero E6 cells at 24 hpi. The viral titers of supernatants from all cells, including parental Vero E6 cells, were saturated at 48 hpi, and most of the cells were detached from the culture dishes at 72 hpi. Furthermore, large syncytia formations in Vero-T2, T11D, T11E, and T13 but not in Vero E6 cells were observed, and these phenomena suggested that the S protein induced cell-to-cell fusion through the activation of these TTSPs (Figure 2E). These results support that TMPRSS11D and 13 enhance cellular entry and subsequent replication of SARS-CoV-2. Although TMPRSS11E did not enhance virus entry at 4 hpi and multicycle replication at 24 hpi in Vero E6 cells, cell fusion activity at 24 hpi was clearly observed. This contradiction may be due to the different efficiencies of fusion caused by TTSPs cleaving the S protein at cell-to-cell and cell-to-virion levels [13,27,37].

## 4. Discussion

In this study, we screened 12 TTSPs and identified TMPRSS11D and 13 as potential proteases which could enhance SARS-CoV-2 entry into cells. Other studies have reported that the SARS-CoV-2 S protein gained cell-to-cell fusion ability in the presence of TMPRSS4, 11A, 11D, and 11E [28,29]. However, these reports were based on findings using cells co-expressing the S protein and TTSPs, and the relationships on the role of TTSPs and native SARS-CoV-2 remained to be elucidated. Our study has demonstrated that the exogenous expressions of both TMPRSS11D and 13 facilitated viral entry into cells and the subsequent replication of SARS-CoV-2.

Consistent with a previous study, TMPRSS4 alone had little effect on SARS-CoV-2 entry into cells [30]. Our TTSP screen by transient transfection identified TMPRSS11E activity to facilitate viral entry in addition to TMPRSS11D and 13 (Figure 1). However, TMPRSS11E did not affect SARS-CoV-2 entry and replication in Vero E6 cells. It has been reported that TMPRSS2 is involved in the entry of coronaviruses not only through the cleavage of the S protein at the S2′ site, but also by other different mechanisms, including S protein cleavage at multiple sites and association with ACE2 [38]. TMPRSS11E may fail to exert viral-entry enhancement in Vero E6 cells due to partial dysfunction of these interactions with the S protein or ACE2. Meanwhile, cell-to-cell fusion was observed in Vero-T11E cells infected with SARS-CoV-2 (Figure 2C–E). The mode of fusion mediated by S protein may be different in cell-to-cell fusion and cell-to-virion fusion [13,27,37]. Collectively, this suggests that the interpretation of cell-to-cell fusion assay, and the role of TMPRSS11E on SARS-CoV-2 infection remains to be clearly established.

Among TTSPs examined in this study, exogenous expression of TMPRSS2 conferred the highest susceptibility among others tested for the entry of SARS-CoV-2 into cells. TMPRSS11D is expressed in the vagina, esophagus, and submucosal serous glands of bronchi and trachea in the human body [39]. TMPRSS13 is predominantly expressed in human skin, lungs, peripheral blood lymphocytes, and certain other glandular epithelium cells [40]. These tissues and cells are not the main target for SARS-CoV-2. It is therefore still unclear whether TMPRSS11D and 13 are involved in the in vivo infection and pathogenicity of SARS-CoV-2. Further studies using in vivo animal models are required to address this question. In conclusion, TMPRSS11D and 13 were identified as potential host serine proteases which can enhance SARS-CoV-2 propagation, and this expands our knowledge on TTSP function and the entry mode of SARS-CoV-2.

## Data Availability

Data sharing is not applicable to this article.

## Supplementary Materials

The following are available online at https://www.mdpi.com/1999-4915/13/3/384/s1, Table S1: Overview of TTSP family gene cloning.
