# Supplementary material for: TMPRSS11D and TMPRSS13 Activate the SARS-CoV-2 Spike Protein
Source: Viruses. 2021 Feb 28;13(3):384. doi: 10.3390/v13030384 (PMC8001073; doi:10.3390/v13030384)
Supplement: Supplementary file 1 [file viruses-13-00384-s001.pdf]

Supplimental Table S1. Overview of TTSP familiy gene cloning.

Subfamily	Protease name	Synonyms	Accession no.	cDNA origin		Sequence of cloning primer* (5'-3')
HAT/DESC	TMPRSS11A	HATL1	NM_182606.4	DNA synthesis	-	-
					-	-
	TMPRSS11D	HAT	NM_004262.3	cDNA clone	F	AAGTCGACGCCACCATGTATAGGCCAGCACGTGTAAC
					R	TAAGCGGCCGCGATCCCAGTTTGTTGCCTAATC
	TMPRSS11E	DESC1	NM_014058.4	cDNA clone	F	AACTCGAGGCCACCATGATGTATCGGCCAGATGTG
					R	TAAGCGGCCGCGATACCAGTTTTTGAAGTAATCCAG
Hepsin/TMPRSS2	TMPRSS1	hepsin, HPN	NM_182983.2	DNA synthesis	-	-
					-	-
	TMPRSS2	Epitheliasin	NM_005656.4	cDNA clone	F	TATCTCGAGACCATGGCTTTGAACTCAGGGTCAC
					R	TATGCGGCCGCGCCGTCTGCCCTCATTTG
	TMPRSS3	TADG-12	NM_024022.3	HepG2 cell	F	ATTCTCGAGCCACCATGGGGGAAAATGATCCGCC
					R	TAAGCGGCCGCGGTTTTTAGGTCTCTCTCCATCTGCTC
	TMPRSS4	CAPH2	NM_019894.4	Caco-2 cell	F	ATTCTCGAGCCACCATGTTACAGGATCCTGACAGTGATCAA
					R	TAAGCGGCCGCCAGCTCAGCCTTCCAGACATTGTAG
	TMPRSS5	Spinesin	NM_030770.4	Calu-3 cell	F	ATTCTCGAGCCACCATGAGCCTGATGCTGGATGACC
					R	TAAGCGGCCGCGAGGAGGGAGTCCTGAGCAGTGTC
	TMPRSS13	MSPL	NM_001077263.3	cDNA clone	F	TAACTCGAGACCATGGAGAGGGACAGCCACGGGA
					R	TATGCGGCCGCGGATTTTCTGAATCGCACCTCG
Matriptase	TMPRSS6	Matriptase-2	NM_153609.4	Hela cell	F	ATTCTCGAGCCACCATGCCCGTGGCCGAGGCCC
					R	TAAGCGGCCGCGGTCACCACTTGCTGGATCCAGCTG
	TMPRSS14	ST14, Matriptase	NM_021978.4	Caco-2 cell	F	ATTCTCGAGCCACCATGGGGAGCGATCGGGCCCG
					R	TAAGCGGCCGCTACCCCAGTGTTCTCTTTGATCCAGTC
Corin	TMPRSS10	Corin	NM_006587.4	MRC5 cell	F	ATTCTCGAGCCACCATGAAACAGTCTCCTGCCCTCGC
					R	TAAGCGGCCGCGTTTAGGAGAAAGGTCTGGATGTAAATC

* Underbars indicate the restriction enzyme site
